# New Composites Derived from the Natural Fiber Polymers of Discarded Date Palm Surface and Pineapple Leaf Fibers for Thermal Insulation and Sound Absorption

**DOI:** 10.3390/polym16071002

**Published:** 2024-04-06

**Authors:** Mohamed Ali, Zeyad Al-Suhaibani, Redhwan Almuzaiqer, Ali Albahbooh, Khaled Al-Salem, Abdullah Nuhait

**Affiliations:** Mechanical Engineering Department, College of Engineering, King Saud University, P.O. Box 800, Riyadh 11421, Saudi Arabia438106677@student.ksu.edu.sa (A.A.); kalsalem@ksu.edu.sa (K.A.-S.);

**Keywords:** date palm surface fibers, pineapple leaf fibers, thermal conductivity coefficient, sound absorption coefficient, agro-waste utilization

## Abstract

New composites made of natural fiber polymers such as wasted date palm surface fiber (DPSF) and pineapple leaf fibers (PALFs) are developed in an attempt to lower the environmental impact worldwide and, at the same time, produce eco-friendly insulation materials. Composite samples of different compositions are obtained using wood adhesive as a binder. Seven samples are prepared: two for the loose natural polymers of PALF and DPSF, two for the composites bound by single materials of PALF and DPSF using wood adhesive as a binder, and three composites of both materials and the binder with different compositions. Sound absorption coefficients (SACs) are obtained for bound and hybrid composite samples for a wide range of frequencies. Flexural moment tests are determined for these composites. A thermogravimetric analysis test (TGA) and the moisture content are obtained for the natural polymers and composites. The results show that the average range of thermal conductivity coefficient is 0.042–0.06 W/(m K), 0.052–0.075 W/(m K), and 0.054–0.07 W/(m K) for the loose fiber polymers, bound composites, and hybrid composites, respectively. The bound composites of DPSF have a very good sound absorption coefficient (>0.5) for almost all frequencies greater than 300 Hz, followed by the hybrid composite ones for frequencies greater than 1000 Hz (SAC > 0.5). The loose fiber polymers of PALF are thermally stable up to 218 °C. Most bound and hybrid composites have a good flexure modulus (6.47–64.16 MPa) and flexure stress (0.43–1.67 Mpa). The loose fiber polymers and bound and hybrid composites have a low moisture content below 4%. These characteristics of the newly developed sustainable and biodegradable fiber polymers and their composites are considered promising thermal insulation and sound absorption materials in replacing synthetic and petrochemical insulation materials in buildings and other engineering applications.

## 1. Introduction

Agro-waste materials are available in huge amounts and are biodegradable, sustainable, eco-friendly, and natural. These wasted materials have to be recycled efficiently; otherwise, they create a burden on the environment. The Organization of Agriculture and Food [1] has reported that Saudi Arabia is one of the largest countries in terms of date production, producing 1.2 million tons/year. The huge amount of waste that can be produced from the date palm trees has many useful applications, such as pulp paper production and as composite materials using fibers [2,3,4]. Furthermore, 20 kg of leaves can be produced from each date palm tree per year as waste. Moreover, atmospheric pollution could occur if such waste is burned in the air, as usually happens as a common practice in some areas of the world [5]. Therefore, good utilization of such wastes will have a good environmental impact in addition to economic benefits. Eleven million tons of agricultural waste per year can be produced in Saudi Arabia, and most of them belong to date palm trees. Those wastes have valuable benefits from an economic point of view [6,7]. Date palm spikelet and date palm fiber have been used with the bricks to improve their thermal insulation characteristics by Belatrache et al. [8]. Their results showed that the thermal conductivity coefficient of the new bricks was 0.106 W/(m K) when 1.36% of date palm fiber was used. Raza et al. [9] have developed a new composite made of date palm surface fiber and polystyrene as a new insulation material for buildings. Their new composite with 20% date palm surface fiber had a low thermal conductivity coefficient of 0.053 W/(m K). Four samples of date palm surface fibers were developed at different densities for thermal insulation by Raza et al. [10] using polyvinyl alcohol as a binder. Their results showed an average thermal conductivity coefficient of 0.038–0.051 W/(m K). Ali and Abdelkareem [11] have reported new thermal insulation materials extracted from date palm surface fibers. The thermal conductivity coefficient range of their produced sample boards was between 0.0475 and 0.0697 W/(m K) using cornstarch resin as a binder. Ali et al. [12] have developed natural insulation materials as a composite between date palm tree leaves and wheat straw fibers. The thermal conductivity of their boards was in the range of 0.045–0.065 W/(m K) at temperatures of 10–60 °C, respectively, using wood adhesive as a binder. Alabdulkarem et al. [13] have developed new experimental thermal insulation materials made as a hybrid between Apple of Sodom fibers and date palm surface fibers with different compositions using wood adhesive, corn starch, and white cement as binders. Their boards had average thermal conductivity coefficients in the range 0.04234–0.05291 W/(m K), and the absorption coefficient of the boards was also determined to be greater than 0.5 at high frequency.

On the other hand, the solid waste, which is a by-product of pineapple industries, is in the range of 40–50% from the peelings, crown, and core (Buckle, [14]). Adhika et al. [15] have reported that the sound absorption coefficient of pineapple fiber with an epoxy composite is greater than 0.5 at high frequencies, and it was affected by the density and applied pressure of the sample. Pineapple leaves were reported as good thermal insulation materials by Tangjuank [16]. He used natural rubber latex as a binder for boards with different densities, and the measured thermal conductivity was in the range of 0.035 W/m. K to 0.043 W/m K. The same binder is used by Kumfu and Jintakosol [17] in producing a thermal insulation board with a density of 338 kg/m^3^ from pineapple leaves with a thermal conductivity coefficient of 0.057 W/(m K). Hybrid of pineapple fibers and polyester using a needle-punching technique was used in developing nonwovens by Thilagavathi et al. [18]. Their product had better thermal insulation and sound-absorbing characteristics compared to pure pineapple fibers. Aerogel composites were made of cotton waste and pineapple leaf by Do et al. [19]. That aerogel was tested as a thermal insulation, and its thermal conductivity coefficient was found in the range of 0.039–0.043 W/(m K). Pineapple leaf fibers with paper waste composites were examined for their sound absorption as an alternative to synthetic fiber by Sari et al. [20]. Their results showed that the sound absorption coefficient increased as the pineapple leaf fibers increased in the composites at the expense of the impact strength. Suphamitmongkol et al. [21] have used pineapple leaf fiber (PALF) as a potential source of sound absorption and thermal insulation materials. They showed that the thermal characteristics of the composites made of PALF were better than those with PET and asbestos but comparable to the composites made of glass fiber. On the other hand, they found that the acoustic properties of PALF are better than those of glass fiber but lower than polyester fiber. Recently, Ali et al. [22] have experimentally studied the effect of using natural polymers of PALF, sunflower seeds, and watermelon seeds and their hybrid composites as new thermal insulation and sound absorption materials. Their results showed that the average thermal conductivity for the composite of PALF and the sunflower seeds was 0.05921 W/(m K) and 0.06577 W/(m K) for the composite of PALF and the watermelon seeds. The sound absorption coefficient was found above 0.5 for most of the bound and hybrid composites. New bio-degradable composite foams made of pineapple stem starch and pineapple leaves were reported by Namphonsane et al. [23]. Their results indicated that the flexural strengths of the composite foams ranged from 1.5 to 4.5 MPa. Furthermore, the sound absorption coefficient of natural fibers such as corn, sugar cane, coir, and dry grass was measured for different thickness samples by Fouladi et al. [24] and found to be good alternatives for common building acoustic boards. Noise reduction and sound absorption coefficients were reported for hemp, kenaf, coconut, cork, sheep wool, cardboard, and cane by Berardi and Iannace [25]. They have shown that these coefficients were thickness, density, and porosity-dependent, and they have been recommended for use in buildings.

Most of the literature mentioned above focused on using natural fiber polymers of DPSF and PALF only; however, bound or hybrid composites were not considered. Therefore, this study presents new novel bound and hybrid composite boards made of date palm surface fibers and pineapple leaf fibers as thermal insulation and sound-absorbing materials. Different densities and composition boards are made, and their thermal conductivity and sound absorption coefficients are found to be promising to use as applications in buildings and can be considered as good biodegrading ecofriendly materials in replacing the synthetic and petrochemical ones.

## 2. Materials and Methods

### 2.1. Collecting the Discarded Materials

**Figure 1:**
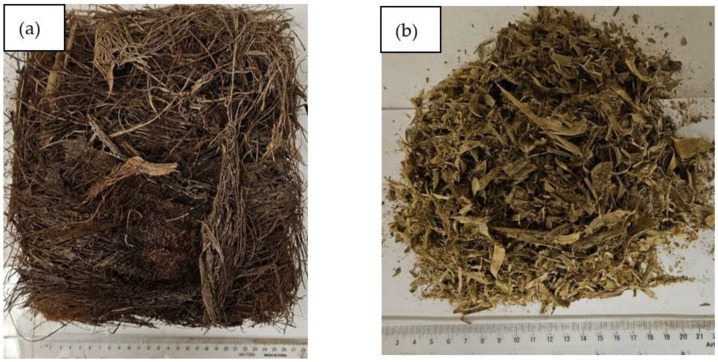
Loose fibers: (**a**) date palm surface fibers (DPSFs) and (**b**) pineapple leaf fibers.

**Figure 2:**
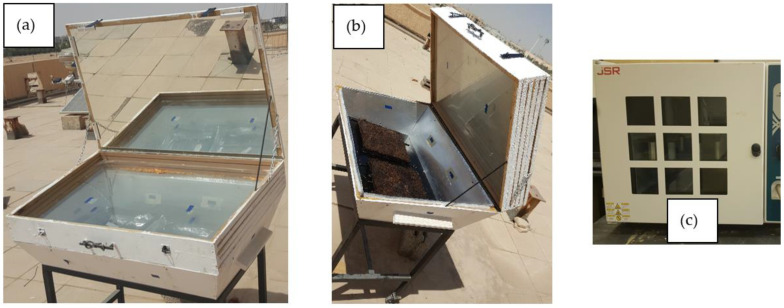
Solar cooker oven ((**a**) closed and (**b**) open) and (**c**) electric convection oven.

At a specified time of the year, the agricultural authority trims a huge amount of date palm trees, wherein they get rid of the residual of such trees, such as date palm surface fibers and leaves. Therefore, those wasted surface fibers are gathered and collected from the authorities before they get rid of them in landfills and create an environmental problem. The pineapple leaves are collected in the same way from the nearby farms in the areas that grow pineapple trees. Another source of pineapple leaves is usually the local juice stores, which get rid of a large amount of the pineapple fruit’s crown, which contains many leaves that are disposed of as waste daily. Figure 1a,b show both the date palm surface fibers and pineapple leaves, respectively. The collected date palm surface fibers are cut to approximately 15 cm in length. The collected pineapple leaves and date palm surface fibers are washed with water to get rid of any dust or other impurities. After that, they are dried either in a covered solar cooker (already exists to save energy) (Figure 2a,b) for sunny days, where the maximum inside temperature reaches 90 °C, or in an electric convection oven at 100 °C, as shown in Figure 2c. The dried natural pineapple leaves are ground in a blender to pieces of an average length of 0.5–3 cm. It should be noted that both natural fiber polymers of DPSF and PALF are used after drying for thermal conductivity measurement with no chemicals or any other treatment. The bound and hybrid composites are moved to the drier after compression in the mold.

### 2.2. Preparing the Samples for Testing

The dried sample boards are prepared in three groups: loose fiber polymers and bound and hybrid composite groups.

#### 2.2.1. Loose Fiber Polymer Group Samples

**Figure 3:**
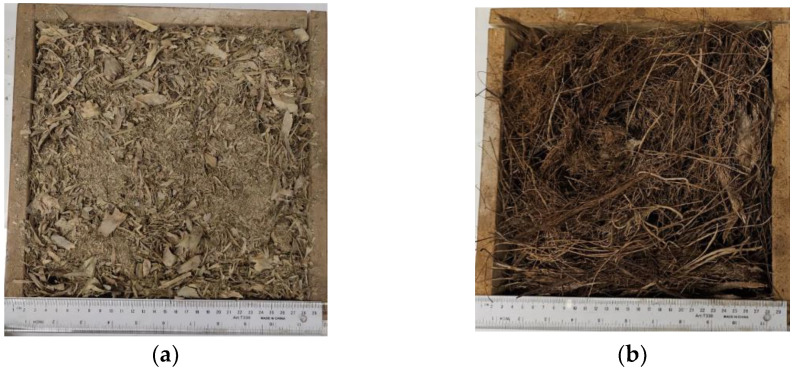
Loose fibers in the wooden mold: (**a**) PALF and (**b**) DPSF.

These groups include the PALF and the DPSF after collecting, washing, and drying them, as described earlier in Section 2.1. The loose fiber polymer samples are enclosed in a wooden mold with inside dimensions of 26.5 × 26.5 × *d* cm^3^, where *d* is the thickness, as shown in Figure 3a,b, to be fitted inside the heat flow meter for thermal conductivity measurement.

#### 2.2.2. Bound Composite Group Boards

**Figure 4:**
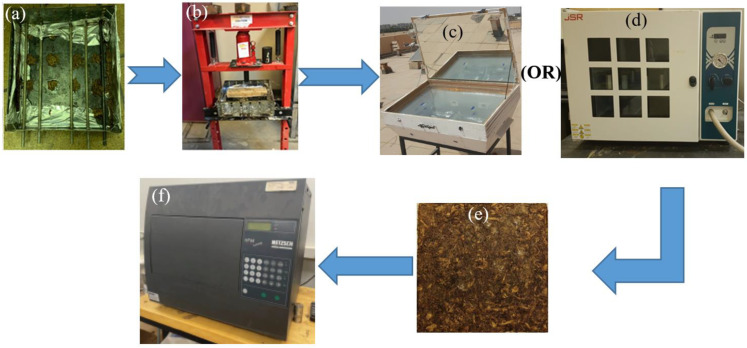
Process of preparing the bound or hybrid polymer samples: (**a**) stainless steel mold holding the sample, (**b**) presser, (**c**) drying solar oven, (**d**) drying electric oven, (**e**) the removed dried sample, (**f**) the heat flow meter for thermal conductivity measurement.

The wood adhesive binder (polyvinyl acetate resin) is used to bind the loose polymer samples. Full specifications, ingredients, and physical and chemical properties of the binder can be found in [26]. A solution of water and wood adhesive is prepared (350 g of the binder and 800 g of water), and the loose leaves or fibers are immersed in it to be sure that each leaf or fiber is in contact with the binder solution. Then, the wetted samples are moved to a stainless mold (Figure 4a), followed by a cold presser to press the sample to a specified size. The load used in pressing the composites is about 173 N, which makes the pressure about 1922 Pa. It should be mentioned that during pressing the composite, the excess solution is discharged; therefore, the actual amount of used adhesive is estimated after the drying process since the mass of the dried fiber is known beforehand. The estimated polymerized mass of the adhesive is introduced in Table 1 with its percentage ratio to the total mass of the composite. After that, the samples are moved either to the solar cooker oven or the electric convection oven for drying, as described in Section 2.1. The dried bound samples are then taken off the mold (Figure 4e) and moved to the heat flow meter for thermal conductivity measurement. Figure 4 summarizes these processes. It should be noted that adding binders to the loose natural polymer fibers increases their thermal conductivity; therefore, one should use the binder, which has a low effect on the thermal conductivity. In our previous study [13], we compared three different binders, namely cornstarch, wood adhesive, and white cement, and the result confirmed that wood adhesive has a lower effect on the thermal conductivity coefficient.

#### 2.2.3. Hybrid Composite Group Boards

**Figure 5:**
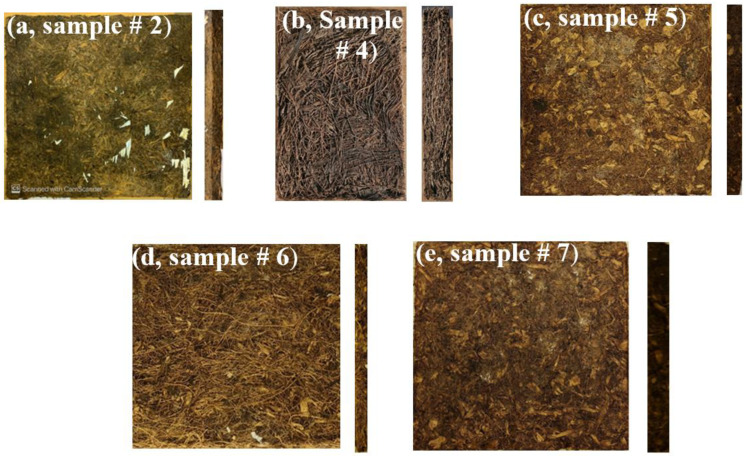
Bound and hybrid composite boards of PALF and DPSF: (**a**) bound composite PALF (# 2), (**b**) bound composite DPSF (# 4), (**c**) hybrid composite of PALF + DPSF (# 5), (**d**) hybrid composite of PALF + DPSF (6), and (**e**) hybrid composite of PALF + DPSF (# 7).

Hybrid composite boards are those samples made of PALF and DPSF by different compositions using the same resin and method described in Section 2.2.2. Figure 5 shows the laboratory-prepared bound and hybrid composite boards. Table 1 shows the complete specification of all developed samples. It should be noted that the bulk density that appears in Table 1 was calculated by measuring the volume of each dried composite (Figure 5) and its mass; then, the density is obtained as the ratio of the mass over the volume. The same procedure was used in calculating the density of the loose polymers, as shown in Figure 3.

## 3. Mechanical Test for Bound and Hybrid Composite Samples

**Figure 6:**
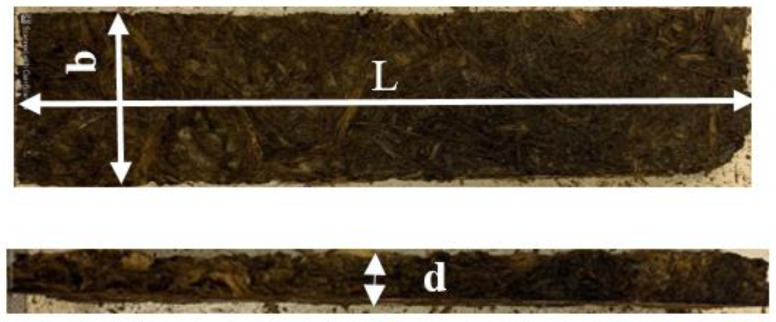
The specimen dimensions used in the three-point flexural test.

The three-point flexural test is obtained for both bound and hybrid composites. Figure 6 shows the specimen used for that test and its dimensions.

The universal testing machine (INSTRON 5984, UTM) with a cross-head of 2 mm/min, which is attached with three points of flexion, is used to perform the flexural test. The flexural stress $\sigma_{f}$ and flexural strain $\epsilon_{f}$ were recorded for each specimen at all applied loads following Equations (1) and (2), respectively, and the flexural modulus $E_{f}$ from Equation (3).

(1) $$\sigma_{f}=\frac{3F\left(L1\right)}{2bd^{2}}$$

(2) $$\epsilon_{f}=\frac{6Dd}{{\left(L1\right)}^{2}}$$

(3) $$E_{f}=\frac{{\left(L1\right)}^{3}m}{4bd^{3}}$$

The software of the machine provides the deflection *D* at each load, where *F*, *L*, *b*, *d*, and *m* are the load (force) at the fracture point, length of the specimen (20 cm), width, thickness, and the gradient (slope) of the initial straight-line portion of the load-deflection curve of the specimen, respectively. Table 2 shows the dimensions of the used specimens. This test follows the ASTM D790-03 standard [27].

## 4. Scanning Electron Microscopy (SEM) Analysis

The surface morphology of the loose, bound, and hybrid composites is determined at different magnifications by using the SEM of type (FE-SEM) (JEOL company, model number JSM7600F, Peabody, MA 01960, USA). A mandatory step before performing the test is to oven-dry the sample first and then coat it with platinum to avoid any electrostatic charging, which may happen during the test. It should be noted that the bound and hybrid composites used for the SEM scanning are obtained from the samples before the mechanical testing, where the objective is focused on the fiber shape and size of the composite and the polymerized binder.

## 5. Thermal Conductivity Coefficient Measurement Test

The heat flow meter (HFM) shown in Figure 4f is used for this test. This HFM is of the bench type (HFM 436 Lambda) manufactured by the German company NETZSCH (NETZSCH-Gerätebau GmbH). This HFM is in accordance with the standards ASTM C518 [28], ISO 8301 [29], JIS A1412 [30], and DIN EN 12667 [31]. The tested samples must have dimensions of 30 × 30 × *d* cm^3^, where *d* is a variable thickness up to 10 cm. The thermal conductivity coefficient for any sample can be determined at any temperature between 20 and 80 °C. Its theory depends on the hot and cold plates, where the heat flows between them at a meat temperature of 20 °C. The thermal conductivity coefficient and temperature accuracy are ±1% to 3% W/(m K) and ±0.01 °C, respectively (manufacturer’s catalog). This HFM is used to determine the thermal conductivity coefficients for either the loose polymer samples shown in Figure 3 or the bound and hybrid composite ones shown in Figure 5. The thermal conductivity coefficient is measured at a wide range of temperatures since the environment temperature in hot weather regions could reach about 45 °C to 50 °C. This wide range of temperatures can also provide the thermal conductivity coefficient dependent on temperature for the new composite materials.

## 6. Sound Absorption Coefficient Test

Sound absorption tests are determined for both bound and hybrid sample numbers 2–7. Two impedance tube sizes are used for a wide range of frequencies: one with a 100 mm diameter for a frequency range of 63–1600 Hz by interchanging the position of the two used microphones and the other with a 30 mm diameter tube for a frequency range of 800–6300 Hz. The software VA-Lab IMP (Ver: V1. 03) was designed by BSWA (BSWA Technology Co., Ltd., Bejing, China), which conforms to ISO 10534-1 [32] and ISO 10534-2 [33] standards. More details and specifications can be found in [34].

## 7. Thermal Stability and Decomposition Test

Thermal stability and decomposition analysis are determined by the thermogravimetric analysis (TGA) and its differential thermogravimetric analyses (DTGAs) for the loose natural polymers of pineapple leaf fibers (PALFs), date palm surface fiber (DPSF), the composite of the bound fibers (# 2 and # 4), and the hybrid composite number 5. Testing Analytical Instrument (TA) TGA Q50 V20.10 Build 36 setup is used. The manufacturer is the Waters Corporation (New Castle, DE 19720, USA). A small amount of each kind of polymer or composite is contained in a platinum pan during the heating up to 600 °C or more. The heating starts at 26 °C at a heating rate of 10 °C/min, and the mass flow rate of the nitrogen gas is 100 mL/min.

## 8. Moisture Content Test

The moisture content test is carried out for the raw loose material of PALF, DPSF, bound composites, and hybrid composites following ASTM D2974-07A [35] standard. Some of each sample is dried in a convection oven (Figure 4d) for one full day, and their mass is denoted as *m*2. The mass is left in the laboratory at a temperature and relative humidity of 21.6 °C and 51.7%, respectively, where their mass is scaled and recorded every five minutes and noted *m*1. The difference between *m*1 and *m*2 presents the moisture content absorbed by the material. This percentage of the absorbed moisture content can be calculated from

(4) $$\%\;\mathrm{of}\;\mathrm{moisture}\;\mathrm{content}=\frac{m1-m2}{m2}\;\times \;100$$

## 9. Results and Discussion

**Figure 7:**
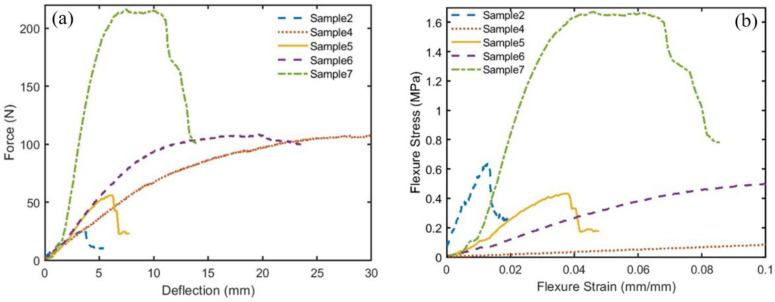
Three-point flexural test results for the bound and hybrid samples: (**a**) force-deflection profiles and (**b**) flexure stress–strain curves.

Figure 7a,b show the force-deflection profiles and the flexure stress–strain curves, respectively, for the bound composite numbers 2 and 4 and the hybrid ones, numbers 5, 6, and 7. The flexural sample’s dimensions are listed in Table 2, and the calculated mechanical properties, such as flexural stress *σ_f_*, flexural strain *ϵ_f_*, and flexural modulus *E_f,_* are shown in Table 3. These parameters are evaluated following Equations (1)–(3) above. Table 3 presents the maximum *σ_f_* before the deviation from linearity [36] (Figure 7b), where the flexural strain *ϵ_f_* is obtained. It should be noted that the slope (m) is calculated from the linear straight line of the profiles in Figure 7a.

**Figure 8:**
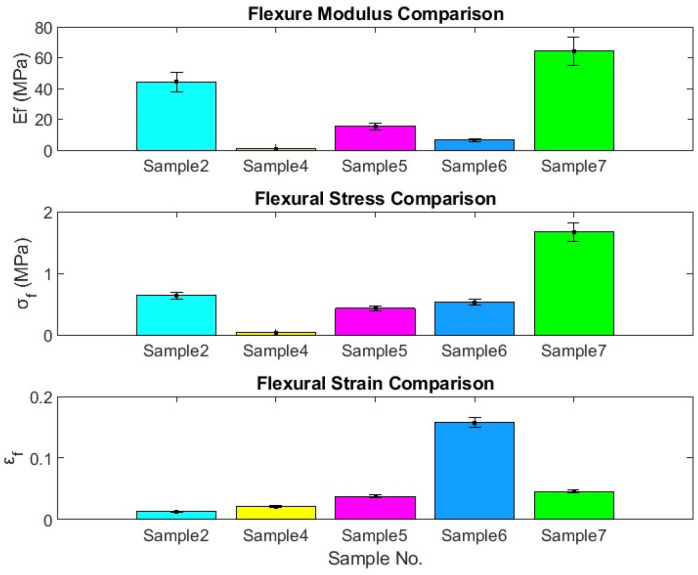
Comparison of the mechanical properties of the bound and hybrid composite samples.

It is worth mentioning that an enhancement is observed in both *E_f_* and *σ_f_* as the density of the specimen increases, which agrees very well with the results obtained by [37,38]. Therefore, specimen number 7 is the best among the hybrid composites, while number 2 is the best among the bound composite specimens. Figure 8 compares the mechanical properties of the hybrid and bound samples in terms of bar charts with error bars. It should be noted that the compactness degree plays a very important role in enhancing the flexural modulus $E_{f}$, flexural stress $\sigma_{f}$, and *ϵ_f_*. This compactness depends on the polymerized binder ratio and the density of the specimen. The error in measuring the length, slope, deflection, and load is ±1.0 mm, ±0.7 N/mm, ±0.001 mm, and ±1.0 N, respectively. A computer program is written to calculate the absolute uncertainty and its percentage following the procedure described by McClintock [39] and Moffat [40]. The maximum uncertainties of flexural modulus (*E_f_*), flexural stress (*σ_f_*), and flexural strain (*ε_f_*) are 13.4%, 8.9%, and 4.6%, respectively.

**Figure 9:**
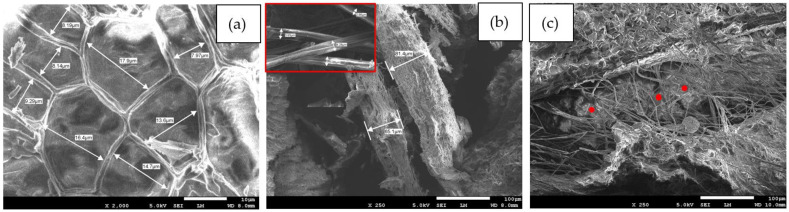
PALF: (**a**) texture of the loose fiber polymers (Lo # 1) at 2000 magnification, (**b**) thickness of the ground leaves, and (**c**) composite (Bo # 2); red spots show the polymerized binder.

Figure 9a,b show a surface morphology comparison of the loose (Lo # 1) and bound composite (Bo # 2) of PALF. Figure 9a shows the texture shape of the loose leaf at 2000 magnification, while Figure 9b shows that after being ground in a blender, where the thickness of the leaves is in the range of 2.86–81.4 μm. Figure 9c shows the composite after mixing and compressing with the binder. Red spots show some of the polymerized binders. It is also noticed that there are a lot of cavities between the skinny fibers, which in turn enhances the thermal conductivity and the sound absorption coefficient, as shown in the next sections.

**Figure 10:**
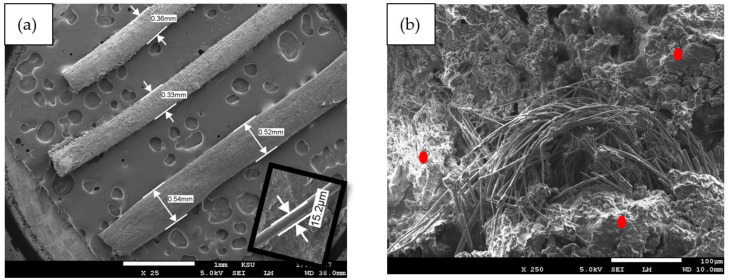
DPSF surface morphology at 250 magnification: (**a**) loose fiber polymers (Lo # 3) at 25 magnification and (**b**) bound composite (Bo # 4) at 250 magnification.

Figure 10a,b show the same configuration but for the DPSF, where Figure 10a shows the size of the loose rough fiber between 15.2 μm and up to 0.54 mm outside diameter. Red spots show the polymerized binders hugging the fibers, leaving some void cavities. It should be noted that the lower corner of Figure 10a shows the small fiber size with larger magnification taken from another photo and montaged here to conserve the number of figures.

**Figure 11:**
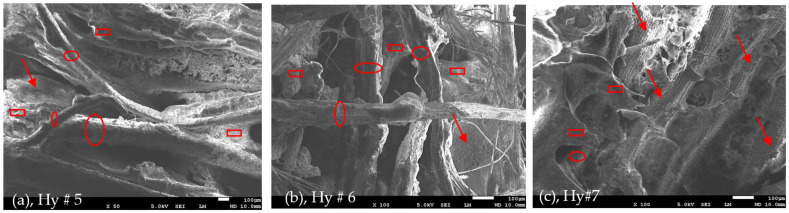
Hybrid composite surface morphology of DPSF and PALF and the binder. (**a**) Hy # 5 composite at 50 magnification, (**b**) Hy # 6 composite at 100 magnification, and (**c**) Hy # 7 at 100 magnification, see text for details.

**Figure 12:**
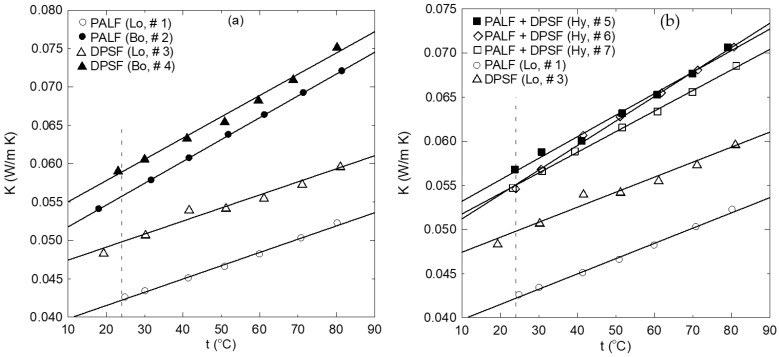
Thermal conductivity coefficient profiles for (**a**) loose and bound samples of DPSF and PALF and (**b**) loose and hybrid samples of DPSF and PALF.

Figure 11a–c show similar surface morphology of the hybrid composite samples 5, 6, and 7, respectively. It should be mentioned that the red arrows, ellipses, and rectangles denoted some of the textures of the PALF, DPSF, and binder, respectively. Figure 12a compares the thermal conductivity coefficient (K) of both loose date palm surface fiber (DPSF, Lo, # 3) and pineapple leaf fibers (PALFs, Lo, # 1) with their bound samples (Bo, # 2 and # 4). Adding binders increases the thermal conductivity coefficient since most of the little porous void spaces in the loose samples get filled with the polymerized binder [22,41,42]. Solid lines present the best curve-fitting through the data. The vertical dashed line at an ambient temperature of 24 °C shows that K for all samples is below 0.06 W/(m K), which indeed promotes these discarded waste polymer and composite materials as good thermal insulation for buildings. Figure 12b compares the loose samples of both fibers to that of hybrid composite numbers 5, 6, and 7 at different compositions, as shown in Table 1. It should be noted that K depends on the amount of polymerized binder used since more binders mean more void porous pores will be filled, which tends to increase K.

Furthermore, the degree of compactness also tends to reduce those pores, which also increases K. In addition, for the same material, increasing the density tends to increase K for the same reason. Moreover, the thermal conductivity depends on the type of materials used. It is also observed that the percentage of increasing the thermal conductivity for the temperature range of 20 °C to 80 °C is 23%, 33%, 23%, 27%, 24%, 29%, and 25% for samples Lo #1, Bo #2, Lo # 3, Bo # 4, Hy #5, Hy # 6, and Hy # 7, respectively. This figure also shows that at an ambient temperature of 24 °C, they have a low thermal conductivity coefficient below 0.06 W/(m K). Solid lines present the linear regression of the data in the form of

K = C1 + C2 t(5)

Table 4 shows the constants C1 and C2 that appear in Equation (4), the coefficient of determination R^2^ of the correlation, the thermal conductivity coefficient at room temperature, and the density of each sample.

**Figure 13:**
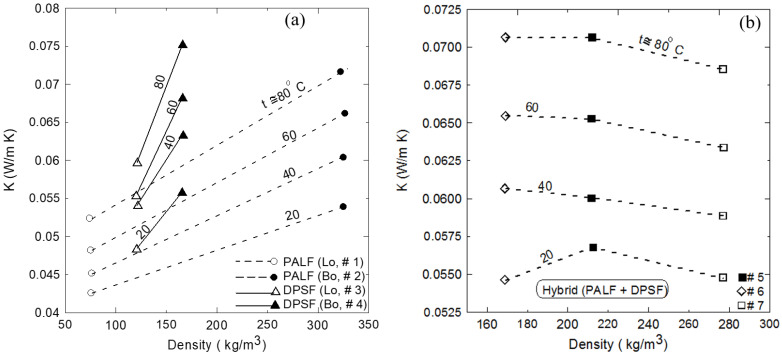
Variation of thermal conductivity coefficients with the density of each sample at different temperatures: (**a**) loose and bound samples and (**b**) loose and hybrid samples.

Figure 13a shows the effect of density on the thermal conductivity coefficient for the same material when it is loose (with no binder) or bound at different temperatures. This figure ensures that for constant density, K increases as the temperature increases. Furthermore, adding a binder (bound composite sample) increases the density and, in turn, increases K at all temperatures. On the other hand, Figure 13b presents the variation of K with the density but for hybrid composite sample numbers 5, 6, and 7 at different temperatures. It should be noted that each curve presents three different samples, each of which may have a different polymerized binder ratio in addition to the different composition of the raw materials at each hybrid composite sample. In this case, the K profile trend looks different than that of Figure 13a due to the different compositions and the ratio of the binder of each sample; therefore, this figure summarizes the relation between K and the density at different temperatures for each hybrid composite sample. Table 5 shows a comparison between the obtained thermal conductivity and those found in the literature for similar materials.

**Figure 14:**
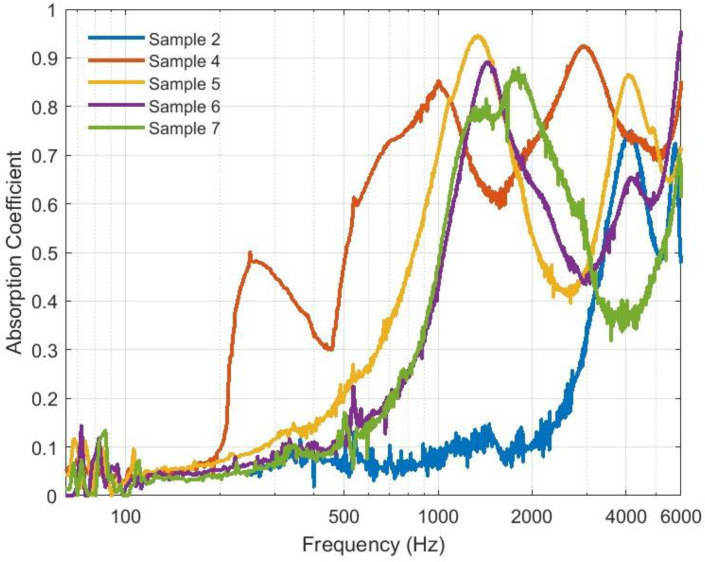
Sound absorption coefficients at a wide range of frequencies for the bound and hybrid samples.

**Figure 15:**
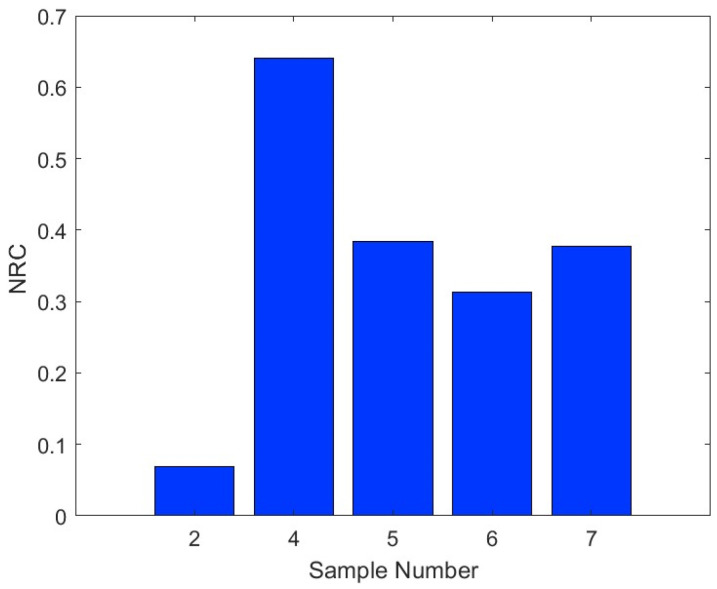
Noise reduction coefficients for bound and hybrid composites.

The low thermal conductivity coefficient of the bound and hybrid composites, which is below 0.07 W/(m K) at all temperature ranges up to 80 °C, promotes their use as insulation materials for buildings and other engineering applications. Figure 14 shows the sound absorption coefficient (SAC) for the bound composite sample numbers 2 and 4 and the hybrid composite ones, numbers 5, 6, and 7, for a frequency range up to 6000 Hz. In the communication range for frequencies up to 2000 Hz, it is noted that hybrid numbers 6 and 7 have SAC greater than 0.4 at a frequency of 1000 Hz and increases until 0.65 at 2000 Hz with a bell shape reaching 0.9 between 1000 and 2000 Hz. Hybrid sample number 5 has an even better SAC at the same frequency range mentioned for the other hybrid ones. On the other hand, the bound sample number 4 has the best SAC in the lower frequency range from 250 to 1000 Hz, which corresponds to SAC in general greater than 0.5. The bound sample number 2 has a lower SAC of about 0.1 up to 2000 Hz. In general, all samples exhibit high SAC at frequencies greater than 2000 Hz. The noise reduction coefficient (NRC) is determined by calculating the average value from the one-third octave values of the SAC at frequencies of 250, 500, 1000, and 2000 Hz and rounding the result to the close 0.05 following [46] and [25], as shown in Table 6. Figure 15 shows a comparison of the NRC of the samples in terms of bar charts. The SAC and the NRC indicate that in the communication range of frequency, the hybrid composite samples, and bound number 4 have good acoustic characteristics, which promote their use as sound-absorbing materials in buildings and other applications. On the other hand, at a frequency greater than 4000 Hz, all samples have good SAC, which means they have the potential to be used for protection against noise emitted by different ultrasonic devices [47]. Moreover, it has been reported [48,49] that materials with SAC ≥ 0.4 can be classified as effective sound-absorbing materials and could be used for absorbing sound in engineering applications.

**Figure 16:**
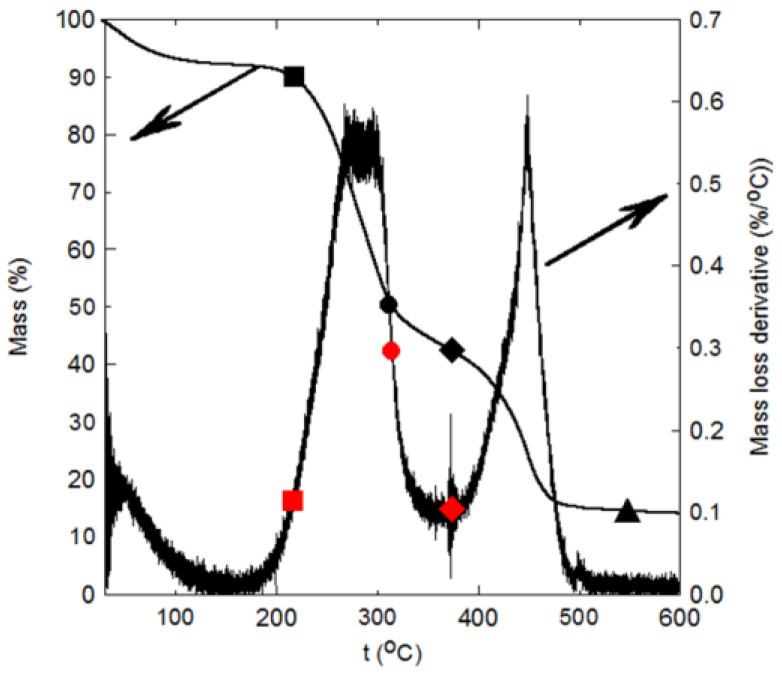
Thermal degradation and decomposition of PALF, see text for details.

Figure 16 shows the profiles of degradation and decomposition of PALF (raw material) through the thermogravimetric analyses (TGAs) and their differential thermogravimetric analysis (DTGA) tests. This figure indicates that the PALF is stable up to about 218 °C, where the material loses its moisture content and its mass decreases by only about 10 percent (■), which corresponds to the starting of its first major degradation (■) in the DTGA profile (left). The material loses 50% of its mass (●), which is shown in the DTGA as (●) at about 315 °C. It is noted that the TGA profile has an inflection point at 372 °C (♦), where the material lost about 58% of its mass with a changing decomposition rate, where the second major degradation starts (♦), as shown on the DTGA profile. The material reaches a char at about 550 °C, where it loses about 86% of its mass (▲). This thermal characteristic and behavior of the PALF indicate that it is thermally stable up to 218 °C, which promotes its suitability for thermal insulation in buildings and other thermal insulation applications.

**Figure 17:**
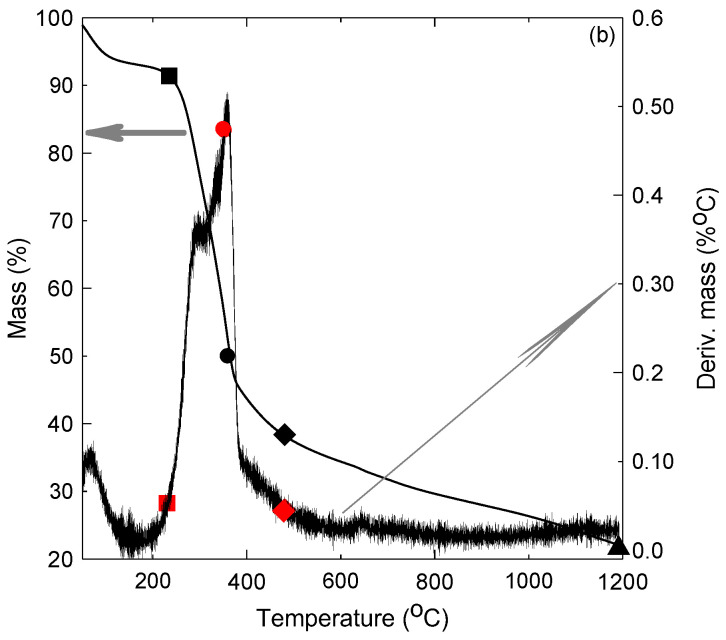
Thermal degradation and decomposition of DPSF, see text for details.

Figure 17 presents the TGA and its DTGA for the date palm surface fibers (DPSFs). This figure indicates that the DPSF is thermally stable up to 232 °C, where the fiber loses about 8.5% of its mass (■). This point is shown as (■) in the DTGA profile, which indicates the start of the major degradation that continues up to 475 °C (♦) or (♦) on the TGA profile. The 50% degradation temperature of the fiber is approximately 364 °C (● on TGA and ● on DTGA), and the fiber reaches a char at about 1192 °C at 22% of its mass (▲). Comparison between Figure 16 and Figure 17 confirms that DPSF is a little more thermally stable than PALF. Nevertheless, both can stand high thermal temperatures above 200 °C.

**Figure 18:**
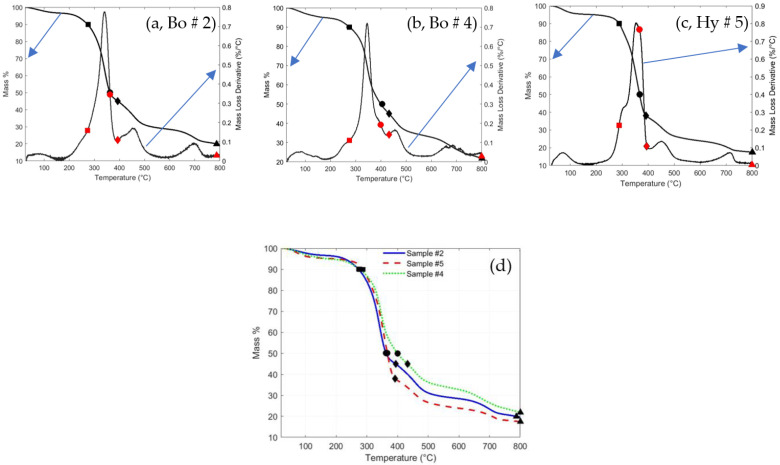
Thermal degradation and decomposition of some composites: (**a**) bound of PALF sample (2), (**b**) bound of DPSF sample (4), (**c**) hybrid of PALF and DPSF sample (5), and (**d**) TGA profiles for the three composites, see text for details.

Figure 18a–c show the TGA and the DTGA for the bound composite sample (2), bound composite sample (4), and hybrid composite sample (5), respectively. They have similar profiles to that of Figure 16 and Figure 17; however, each composite has its own stability, degradation, and char formation temperature, as shown in Table 7. Figure 18d shows a comparison of the TGA profiles of the bound and hybrid composites. Table 7 indicates that the bound or hybrid composites are more thermally stable than the loose fiber polymers since their thermally stable temperatures are 272.8 °C, 275.8 °C, and 287.8 °C for sample numbers 2, 4, and 5, respectively, higher than that for the loose PALF and DPSF. This observation is due to the binders.

**Figure 19:**
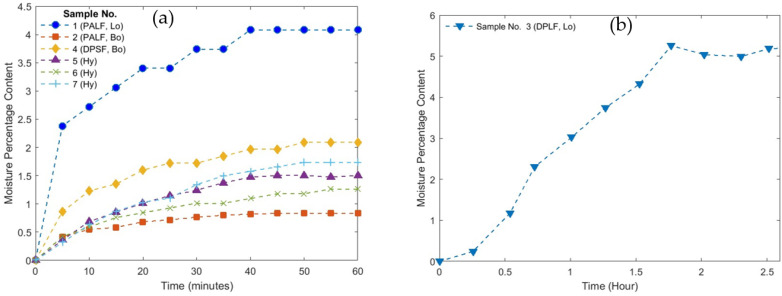
Moisture content profiles. (**a**) Samples 1, 2, 4, 5, 6, and 7 and (**b**) loose DPSF sample 3.

**Figure 20:**
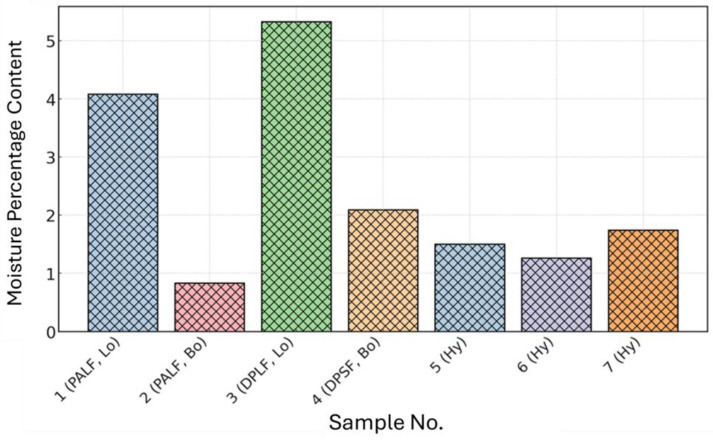
Moisture content profile for all samples at steady state conditions.

The bound and hybrid composites are thermally stable at higher temperatures above 200 °C, which gives them the potential to be used as safe insulation materials for buildings and other engineering applications. Figure 19a shows the moisture content profiles for the loose PALF (Lo # 1) polymer, bound (Bo # 2), bound of DPSF (Bo # 4), and hybrid composite sample numbers 5, 6, and 7 until they reach a steady state condition. This figure ensures that the PALF has a low percentage of about 4% moisture content. It is also observed that all the bound and hybrid composites have much lower moisture content (less than 2%) since most of the void porous spaces of the loose fibers are filled by the binder and hence reduce their ability to absorb more moisture. Therefore, these low moisture contents are much below the 16% that presents safe moisture content, as suggested by Bainbridge [50] for similar natural straw fibers. The moisture content for the loose DPSF polymer (Lo # 3) is presented in Figure 19b since it reaches a steady state at a longer time of about 2.5 hours. Figure 20 shows a bar chart of moisture content percentage for all samples at steady-state conditions for easier comparison.

## 10. Conclusions

New hybrid polymers and composite thermal insulation and sound absorption materials were made of date palm surface fibers (DPSFs) and pineapple leaf fibers (PALFs) using wood adhesive as a binder. The result of the thermal conductivity coefficients of the samples are very optimistic and in the range of 0.042–0.06 W/(m K), 0.052–0.075 W/(m K), and 0.054–0.07 W/(m K) for the loose fiber polymers and bound and hybrid composites, respectively. The bound composite of DPSF has very good acoustic characteristics, such as a noise reduction coefficient (NRC) of 0.64 and sound absorption coefficient (SAC) greater than 0.5 for frequencies greater than 250 Hz, followed by the hybrid composites, as shown in Table 6 and Figure 14. Both raw materials of PALF and DPSF are thermally stable up to 218 °C and 232 °C, respectively. All bound and hybrid composites are thermally stable at temperatures higher than 270 °C, as shown in Table 7. Most of the bound and hybrid samples have mechanical properties such as a flexure modulus in the range of 6.47–64.16 MPa and a flexure stress range of 0.43–1.67 MPa. The loose PALF and DPSF have very low moisture contents of about 4% and 5%, respectively, and the bound composite of PALF and DPSF has a moisture content of less than 1% and about 2%, respectively. All other hybrid composites have less than 2% moisture content. These new samples of thermal insulation and sound absorption materials, either bound or hybrid, are natural, biodegradable, eco-friendly, and sustainable, which promotes them as possible replacements for synthetic and petrochemical materials in building construction and other engineering applications.

## Figures and Tables

**Table 1:** Physical properties and dimensions of the developed polymer samples.

**Material**	**Sample Number**						
	**Lo (# 1)**	**Bo (# 2)**	**Lo (# 3)**	**Bo (# 4)**	**Hy (#5)**	**Hy (#6)**	**Hy (# 7)**
DPSF %	0.0	0.0	100	75.9	46.0	33.0	18.0
Mass of DPSF (g)	0.0	0.0	500	500.2	272.3	150.15	107.6
PALF %	100	87	0.0	0.0	15.0	33.0	53.0
Mass of PALF (g)	106	321.9	0.0	0.0	88.8	150.15	317.0
The ratio of the polymerized binder to the total mass %	0.0	13	0.0	24.1	39.0	34.0	29.0
Mass of the binder (g)	0.0	48.1	0.0	158.8	230.9	154.7	173.4
Thickness, (mm)	21.0	13.0	46.0	44.0	30.0	30.0	24.0
Figure #	3a	5a	3b	5b	5c	5d	5e
Density of dried specimen (kg/m^3^)	76.5	329	121	166	212	169	277
Total dried mass (g)	106	370	500	659	592	455	598

**Table 2:** Dimensions of the flexural specimens.

Specimen No.	Thickness *d* (mm)	Width *b* (mm)	Span (*L*1) (mm)
2	13.5	49.5	150
4	43	59.50	130
5	23	55.0	150
6	30	51.0	150
7	23	55.0	150

**Table 3:** Mechanical properties of the three-point flexural tests.

Specimen No.	Slop, m (N/mm)	Flexure Modulus (MPa), *E_f_*	Density, kg/m^3^	Flexural Stress (MPa), *σ_f_*	Flexural Strain at Flexural Strength, *ϵ_f_*
2	6.4	44.2 ± 5.9	329	0.64 ± 0.057	0.01 ± 0.001
4	6.5	0.8 ± 0.1	166	0.04 ± 0.003	0.02 ± 0.001
5	12.3	15.4 ± 2.1	212	0.43 ± 0.039	0.04 ± 0.002
6	10.6	6.5 ± 0.9	169	0.53 ± 0.047	0.16 ± 0.007
7	50.9	64.2 ± 8.6	277	1.67 ± 0.149	0.05 ± 0.002

**Table 4:** Constants and R^2^ for correlation (1) and K at ambient temperature.

Sample Number	C1	C2	R^2^ (%)	K at 24 °C	Density, kg/m^3^
1 (PALF, Lo)	0.038	0.00017	99.6	0.0425	76.5
2 (PALF, Bo)	0.049	0.00028	99.9	0.0557	329
3 (DPSF, Lo)	0.046	0.00017	97.2	0.0498	121
4 (DPSF, Bo)	0.052	0.00028	99.1	0.0592	166
5(Hy)	0.051	0.00024	99.1	0.0568	212
6(Hy)	0.048	0.00028	99.6	0.0546	169
7(Hy)	0.049	0.00023	99.8	0.0547	277

**Table 5:** Range of thermal conductivity coefficients of similar materials in the literature and comparison with the ones obtained from the current study.

Polymer Fibers or Composites	Density (kg/m^3^)	Thermal Conductivity (w/mk)	References
PALF (Bo #2)	329	0.0541–0.0721	Current
DPSF (Bo # 4)	166	0.05918–0.075302	Current
Hy (# 5)	212	0.05679–0.070622	Current
Hy (# 6)	169	0.054595–0.07065	Current
Hy (#7)	277	0.054717–0.068542	Current
Bound sunflower seed fibers	248	0.0617–0.0801	[22]
Bound watermelon	472	0.0669–0.0982	[22]
Bound eucalyptus globulus leaves	153.0	0.0472–0.0599	[41]
Bound wheat straw fibers	130.0	0.0466–0.0569	[41]
The hybrid of eucalyptus globulus leaves and wheat straw fibers	211.0	0.0460–0.0574	[41]
Hybrid (date palm surface fibers + Apple of Sodom fibers)	114.0–233.0	0.0423–0.0529	[13]
Date palm surface fibers	176–260	0.0475–0.0697	[11]
Bagasse	70–350	0.0460–0.0550	[43]
Straw bale	50–150	0.0380–0.0670	[43]
Rice husk	154–168	0.0464–0.566	[43]
Corn cob	171–334	0.101	[44]
Jute	26.1	0.0458	[44]
Flax	32.1	0.0429	[44]
Technical hemp	30.2	0.0486	[44]
Coconut fiber	40–90	0.0480–0.0576	[45]
Kenaf	30–180	0.034–0.043	[43]

**Table 6:** Density, sound absorption coefficients (SACs) at one-third octave values, and noise reduction coefficients (NRCs).

Sample Number	Density, kg/m^3^	Frequency (Hz)				NRC
		250 Hz	500 Hz	1000 Hz	2000 Hz	
		Sound Absorption Coefficients (SACs)				
2	329	0.047	0.073	0.054	0.101	0.069
4	166	0.501	0.482	0.853	0.725	0.641
5	212	0.087	0.213	0.704	0.534	0.384
6	169	0.066	0.113	0.432	0.640	0.313
7	277	0.050	0.171	0.471	0.816	0.377

**Table 7:** Thermally stable, T_50_% degradation, inflection, and char formation temperature for the bound and hybrid composites, as shown in Figure 18.

Bound Composite Sample 2 (Figure 18a)							
Thermally Stable		T_50_% Degradation		Inflection		Char Formation	
Mass % 90.0	Temp. (°C) 272.8	Mass % 50.0	Temp. (°C) 362.6	Mass % 45.0	Temp. (°C) 393.8	Mass % 20.0	Temp. (°C) 787.0
Hybrid composite sample 5 (Figure 18c)							
Thermally stable		T_50_% degradation		inflection		Char formation	
Mass % 90.0	Temp. (°C) 287.8 °C	Mass % 50.0	Temp. (°C) 366.8	Mass % 38.0	Temp. (°C) 391.7	Mass % 15.0	Temp. (°C) 800.0
Bound composite sample 4 (Figure 18b)							
Thermally stable		T_50_% degradation		inflection		Char formation	
Mass % 90.0	Temp. (°C) 275.8	Mass % 50.0	Temp. (°C) 400.0	Mass % 45.0	Temp. (°C) 432.5	Mass % 20.0	Temp. (°C) 800

## Data Availability

The raw data supporting the conclusions of this article will be made available by the authors upon request.
